# Activation of Protein Kinase A and Exchange Protein Directly Activated by cAMP Promotes Adipocyte Differentiation of Human Mesenchymal Stem Cells

**DOI:** 10.1371/journal.pone.0034114

**Published:** 2012-03-27

**Authors:** Bingbing Jia, Lise Madsen, Rasmus Koefoed Petersen, Nathalie Techer, Reidun Kopperud, Tao Ma, Stein Ove Døskeland, Gérard Ailhaud, Jinfu Wang, Ez-Zoubir Amri, Karsten Kristiansen

**Affiliations:** 1 Institute of Cell Biology and Genetics, College of Life Sciences, Zhejiang University, Hangzhou, People's Republic of China; 2 Department of Biology, University of Copenhagen, Copenhagen, Denmark; 3 National Institute of Nutrition and Seafood Research, Bergen, Norway; 4 IBV, Université de Nice Sophia-Antipolis, UMR7277 CNRS - UMR1091 INSERM, Faculté de Médecine, Nice, France; 5 Department of Biomedicine, University of Bergen, Bergen, Norway; Universidade Federal do Rio de Janeiro, Brazil

## Abstract

Human mesenchymal stem cells are primary multipotent cells capable of differentiating into several cell types including adipocytes when cultured under defined *in vitro* conditions. In the present study we investigated the role of cAMP signaling and its downstream effectors, protein kinase A (PKA) and exchange protein directly activated by cAMP (Epac) in adipocyte conversion of human mesenchymal stem cells derived from adipose tissue (hMADS). We show that cAMP signaling involving the simultaneous activation of both PKA- and Epac-dependent signaling is critical for this process even in the presence of the strong adipogenic inducers insulin, dexamethasone, and rosiglitazone, thereby clearly distinguishing the hMADS cells from murine preadipocytes cell lines, where rosiglitazone together with dexamethasone and insulin strongly promotes adipocyte differentiation. We further show that prostaglandin I_2_ (PGI_2_) may fully substitute for the cAMP-elevating agent isobutylmethylxanthine (IBMX). Moreover, selective activation of Epac-dependent signaling promoted adipocyte differentiation when the Rho-associated kinase (ROCK) was inhibited. Unlike the case for murine preadipocytes cell lines, long-chain fatty acids, like arachidonic acid, did not promote adipocyte differentiation of hMADS cells in the absence of a PPARγ agonist. However, prolonged treatment with the synthetic PPARδ agonist L165041 promoted adipocyte differentiation of hMADS cells in the presence of IBMX. Taken together our results emphasize the need for cAMP signaling in concert with treatment with a PPARγ or PPARδ agonist to secure efficient adipocyte differentiation of human hMADS mesenchymal stem cells.

## Introduction

Obesity is a prevalent condition frequently associated with metabolic disorders such as dyslipidemia, diabetes, hypertension and cardiovascular diseases. Fat mass excess in severe obesity is typically not only due to an increase in adipocyte size, but also adipocyte number [1], [2]. *In vivo* studies in both human and rodents have illustrated that high fat diets, especially when combined with carbohydrates, promote both hyperplasic and hypertrophic development of adipose tissue [3]–[8]. Of note, results from animal feeding studies have indicated that the effect of dietary fat on adipose tissue mass, at least in part, depends on the source and nature of the fatty acids. Compared to saturated fatty acids, *n*-3 polyunsaturated fatty acids (PUFAs) have been reported to be less adipogenic and to prevent excessive growth of adipose tissue in rodents [9]–[11]. The effect of *n*-6 PUFAs on adipogenesis and obesity has been controversial as *n*-6 PUFAs, including arachidonic acid, have been reported to be both pro- and anti-adipogenic, both *in vitro* and *in vivo* [6], [12], [13]. However, we have recently shown that the background diet, in particular the ratio between carbohydrate and protein, acts on insulin secretion and cAMP signaling determine the adipogenic action of both *n*-3 and *n*-6 PUFAs, explaining at least in part the apparently discrepant reports on the effect of PUFA supplementation [6]–[8], [14].

Adipocytes are derived from mesenchymal stem cells (MSCs) [15] which have the potential to differentiate into several lineages including adipocytes, osteoblasts, chondroblasts, skeletal muscle cells and neurocytes [16]–[18]. Under *in vitro* conditions, adipocyte differentiation of MSCs can be induced by a hormonal cocktail which includes insulin, glucocorticoid and cAMP elevating agents that together activate several signaling pathways culminating in the activation of PPARγ and terminal adipocyte differentiation [15]. Of note, in contrast to murine preadipocytes, differentiation of human preadipocytes requires the addition of a PPARγ agonist [19].

In addition to PPARγ, PPARδ and PPARα have also been implicated in adipocyte differentiation [20]–[22]. PPARs are activated by fatty acids and fatty acid-derived metabolites including leukotrienes, prostaglandins, and lipoxygenase products [23]–[27].The *n*-6 PUFA arachidonic acid has been identified as one of the main adipogenic fatty acid components of serum [28]. Arachidonic acid can act by direct binding to PPARs, but can also be converted into various derivatives which stimulate differentiation by activating PPARs [29], [30]. Apart from serving as a PPAR ligand or a ligand precursor, arachidonic acid may promote adipocyte differentiation via transformation into prostacyclin (PGI_2_) that is known to elevate intracellular cAMP levels and thereby promote terminal differentiation of the two murine preadipocytes cell lines Ob1771 and 3T3-F442A [31]. However, it should be noted that the effect of exogenously added arachidonic acid in the commonly used 3T3-L1 cell differentiation model depends on the simultaneous presence of a cAMP-elevating agent [6], [26].

Most intracellular effects of cAMP are mediated by the protein kinase A (PKA) [32] and the exchange protein directly activated by cAMP (Epac), a guanine nucleotide exchange factor activated by cAMP [33], [34]. The Epac protein family comprises two main isoforms, Epac1 and Epac2, which are also known as RAPGEF3 and RAPGEF4, respectively. Each contains a functional cAMP-binding domain and a guanine nucleotide-exchange factor domain, which activates the small GTPase Rap1 in response to cAMP [35], [36]. The functional analysis of Epac was greatly facilitated by the development of the cAMP analog 8-pCPT-2′O-Me-cAMP, which binds specifically to Epac1 and Epac2 with no significant activation of other cAMP targets, i.e. PKA and ion channels [37]. Epac has been implicated in the regulation of several important biological functions, including insulin secretion, cell adhesion, neural differentiation, proliferation and ion transport [38], [39]. Rho GTPases are essential components of the signaling pathway that directs reorganization of the actin cytoskeleton. The action of Rho is mediated by the downstream effector, Rho-associated kinase (ROCK) [40], [41]. Rho A and ROCK have been shown to play pivotal roles during commitment of MSCs by modulating cell shape and cytoskeletal tension [42], [43].

We have recently reported that Epac via Rap1 and PKA synergistically promote adipogenesis of 3T3-L1 cells [44]. We provided evidence that Epac activation promotes adipocyte differentiation by counteracting the decrease in IGF-1/insulin signaling mediated via an efficient PKA-dependent inhibition of Rho/ROCK activity [45]. Accordingly, we demonstrated that Epac activation was sufficient to enhance adipocyte differentiation when ROCK was inhibited.

In contrast to the situation prevailing in mouse preadipocytes, the role of Epac for induction of adipocyte differentiation of human adipocyte precursor cells is still poorly understood. In the present study we demonstrate that adipocyte differentiation *in vitro* of human mesenchymal stem cells derived from adipose tissue (hMADS cells), is critically dependent of cAMP signaling requiring activation of both the PKA and the Epac branch of signaling. Furthermore, consistent with the results obtained with the murine 3T3-L1 cells, selective activation of Epac robustly induced adipocyte differentiation of hMADS cells when ROCK activity was inhibited. Of note, and contrasting murine preadipocytes, effective differentiation of hMADS cells even in the presence of the powerful PPARγ agonist rosiglitazone required inclusion of a cAMP elevating agent. We evaluated thoroughly the effects of PPAR agonists and various fatty acids on adipocyte differentiation of hMADS cells. Contrasting previous observation using murine preadipocytes, none of the tested fatty acid, including arachidonic acid, promoted adipocyte differentiation of hMADS cells. Interestingly, we found that prolonged treatment with the synthetic PPARδ agonist L165041 also promoted adipocyte differentiation of hMADS cells in the presence of the cAMP elevating agent isobutylmethylxanthine (IBMX). Taken together our results emphasize the need for cAMP signaling in concert with treatment with a PPARγ or PPARδ agonist to secure efficient adipocyte differentiation of human hMADS mesenchymal stem cells.

## Results

### Elevated levels of cAMP promote adipocyte differentiation of hMADS cells

**Figure 1:**
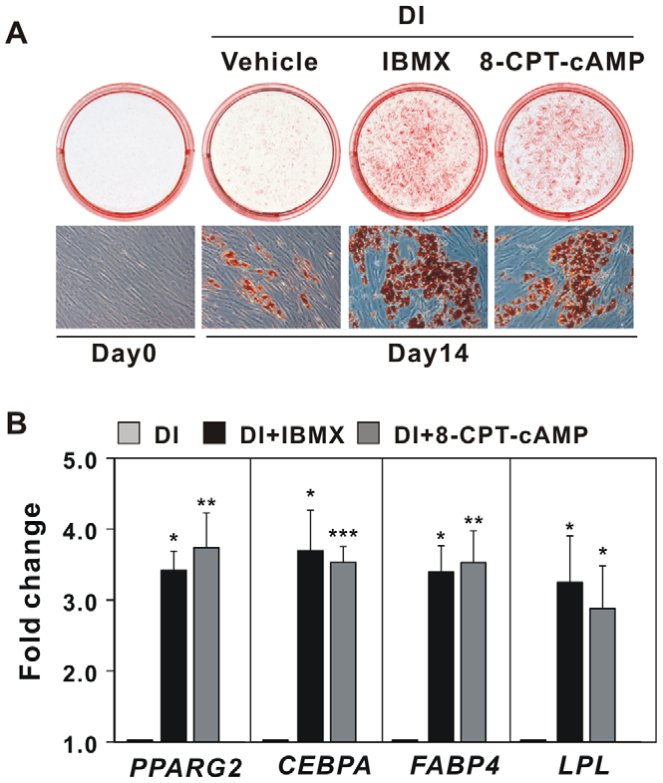
A cAMP elevating agent is required for adipocyte differentiation of hMADS cells. (A) Two-day post-confluent hMADS cells were induced to differentiate with 1 µM Dex and 0.86 µM insulin (DI) in the presence or absence of 0.5 mM IBMX, 100 µM 8-pCPT-cAMP or an equal volume of water (vehicle) from day 0 to day 3. Thereafter, 0.5 µM rosi was added until day 9. On day 14, cells were fixed, and cytoplasmic triglycerides were stained with Oil-Red-O. Undifferentiated hMADS cells were used as control. The photographs and micrographs shown are representative of 3 independent experiments. (B) RNA was isolated on day 14, and expression of *PPARG2*, *CEBPA*, *FABP4* and *LPL* was determined by RT-qPCR. Data presented were normalized to the value of DI treated cells. Significant differences relative to DI treated cells are indicated by asterisks, *p<0.05, **p<0.01, ***p<0.001, n = 6.

To determine whether elevated levels of intracellular cAMP also are required to promote adipocyte differentiation of hMADS cells, two-days-post-confluent hMADS cells were treated with the adipogenic cocktail with or without a cAMP elevating agent. Oil-Red-O staining and adipocyte marker gene expression clearly demonstrated that elevation of cAMP levels by addition of the phosphodiesterase inhibitor IBMX or the cell-permeable cAMP analog 8-pCPT-cAMP strongly enhanced adipocyte differentiation. In the absence of elevated levels of cAMP only very few preadipocytes underwent differentiation even in the presence of the strong inducers of adipocyte differentiation, insulin, dexamethasone (Dex) and rosiglitazone (Fig. 1A and 1B).

### The adipogenic effect of cAMP requires the activation of both PKA- and Epac-dependent signaling pathways

**Figure 2:**
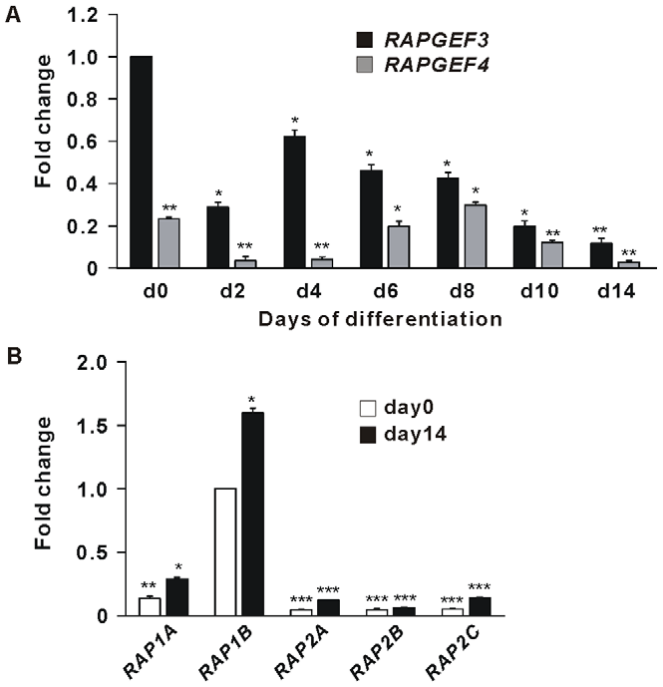
Expression of *Epac* and *Rap* mRNAs in hMADS cells. Cells were differentiated as described in “Materials and Methods”. RNA was isolated on the indicated days. The levels of *RAPGEF3 (Epac1)*, *RAPGEF4 (Epac2)* mRNAs (A) and *RAP1A*, *RAP1B*, *RAP2A*, *RAP2B*, *RAP2C* mRNAs (B) were determined by RT-qPCR. Data presented in (A) were normalized to *RAPGEF3* on day 0; in (B) data were normalized to *RAP1B* on day0. Significant differences are indicated by asterisks, *p<0.05, **p<0.01, ***p<0.001, n = 8.

**Figure 3:**
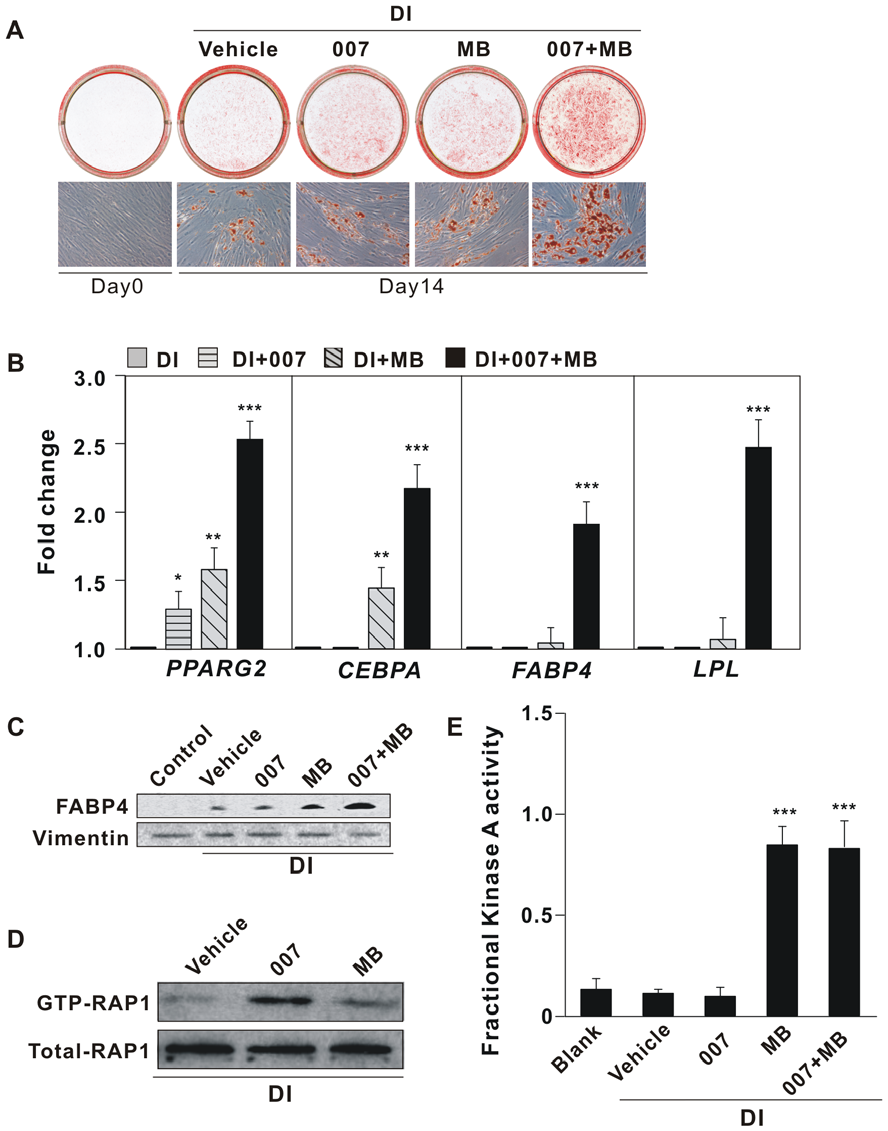
Adipocyte differentiation of hMADS cells requires the activation of both Epac- and PKA-dependent signaling pathways. Two-day post-confluent hMADS cells were induced to differentiate with 1 µM Dex, 0.86 µM insulin (DI), in the presence of 200 µM of the Epac-selective cAMP analog 8-pCPT-2′-O-Me-cAMP (007) [37], 100 µM of PKA selective cAMP analog 6-MB-cAMP (MB) [37], either separately or in combination (007+MB) from day 0 to day 3. Rosi (0.5 µM) was present from day 3–9. Undifferentiated hMADS cells were taken as control. (A) The panel shows cells on day 14 stained with Oil-Red-O. The photographs and micrographs shown are representative of 3 independent experiments. (B) RNA was isolated on day 14, and expression of *PPARG2*, *CEBPA*, *FABP4* and *LPL* was determined by RT-qPCR. Date presented were normalized to the value of DI treated cells. Significant differences are indicated by asterisks, *p<0.05, **p<0.01, ***p<0.001, n = 9. (C) Whole cell extracts were prepared and analyzed for FABP4 protein level by Western blotting. (D and E) Two-day post-confluent hMADS cells were treated for 15 min with 200 µM 8-pCPT-2′-O-Me-cAMP (007) or 100 µM 6-MB-cAMP (MB) or an equal volume of water (vehicle) in medium with Dex and Insulin. (D) GTP bound RAP1 was measured by a RAP1 activation pull-down assay as described in “Materials and Methods”. (E) PKA activity in cell lysates was determined as described in “Materials and Methods”. Significant differences relative to DI treated cells are indicated by asterisks, *p<0.05, **p<0.01, ***p<0.001, n = 4.

We have recently demonstrated that cAMP-stimulated differentiation of murine 3T3-L1 cells into adipocytes *in vitro* requires Epac/Rap signaling [44]. Given the importance of a cAMP-elevating agent during differentiation of hMADS cells in serum-free conditions, we sought to determine the requirement of Epac/Rap in these human cells. Real-time qPCR analyses demonstrated that both *RAPGEF3 (Epac1)* and *RAPGEF4 (Epac2)* mRNA were expressed in hMADS cells (Fig. 2A). Expression of *RAPGEF3* mRNA, the predominant isoform, transiently decreased after induction of differentiation, followed by increased expression on day 4. After day 4, *RAPGEF3* mRNA level declined gradually to barely detectable levels in mature adipocytes (day 14). By contrast, expressions of *RAP1A* and *RAP1B* mRNA (the dominant Rap isoforms in hMADS cells) were higher in mature adipocytes than in undifferentiated cells (Fig. 2B). Having established that *Epac* and *RAP1* are expressed in hMADS cells, we tested whether the selective Epac-activating cAMP-analog 8-pCPT-2^′^-*O*-Me-cAMP (007), alone or in combination with the selective PKA-activating analog 6-MB-cAMP (MB), could mimic the effect of IBMX in stimulating adipogenesis. In accordance with results obtained in murine preadipocytes [44], addition of 007 or MB alone only slightly enhanced adipocyte differentiation of hMADS cells. However, as demonstrated by Oil-Red-O staining, a majority of the cells rounded up to become lipid-filled adipocytes when treated with both 007 and MB (Fig. 3A). The robust induction of adipocyte marker genes mRNAs (Fig. 3B) and proteins (Fig. 3C) in cells treated with both 007 and MB confirmed the adipocyte phenotype. The selectivity of 007 and MB on Epac and PKA activation, respectively, was confirmed by measurement of Rap activation and fractional PKA activity. As predicted, treatment of hMADS cells with 007, but not MB, led to activation of Rap1 as determined by increased levels of GTP-loaded Rap1 (Fig. 3D). *Vice versa*, treatment of the hMADS cells with MB alone or in combination with 007 increased fractional PKA activity, whereas 007 alone had no effect (Fig. 3E). We further assessed the effects of the PKA-specific inhibitors, the cAMP analogs Rp-8-CPT-cAMPS and Rp-8-Br-cAMPS (Rps) [37], [44] on adipocyte differentiation of hMADS cells induced by DI+007+MB (Fig. S1). Addition of Rps decreased the triglyceride content in the cells (Fig. S1A) and significantly reduced the induction of adipocyte marker genes mRNAs (Fig. S1B), emphasizing the importance for PKA activity for adipocyte differentiation of hMADS cells. Together, these results indicate that the adipogenic effect of cAMP requires both Epac and PKA activity in hMADS cells.

### Epac1 is required for isobutylmethylxanthine-induced adipogenesis of hMADS cells

**Figure 4:**
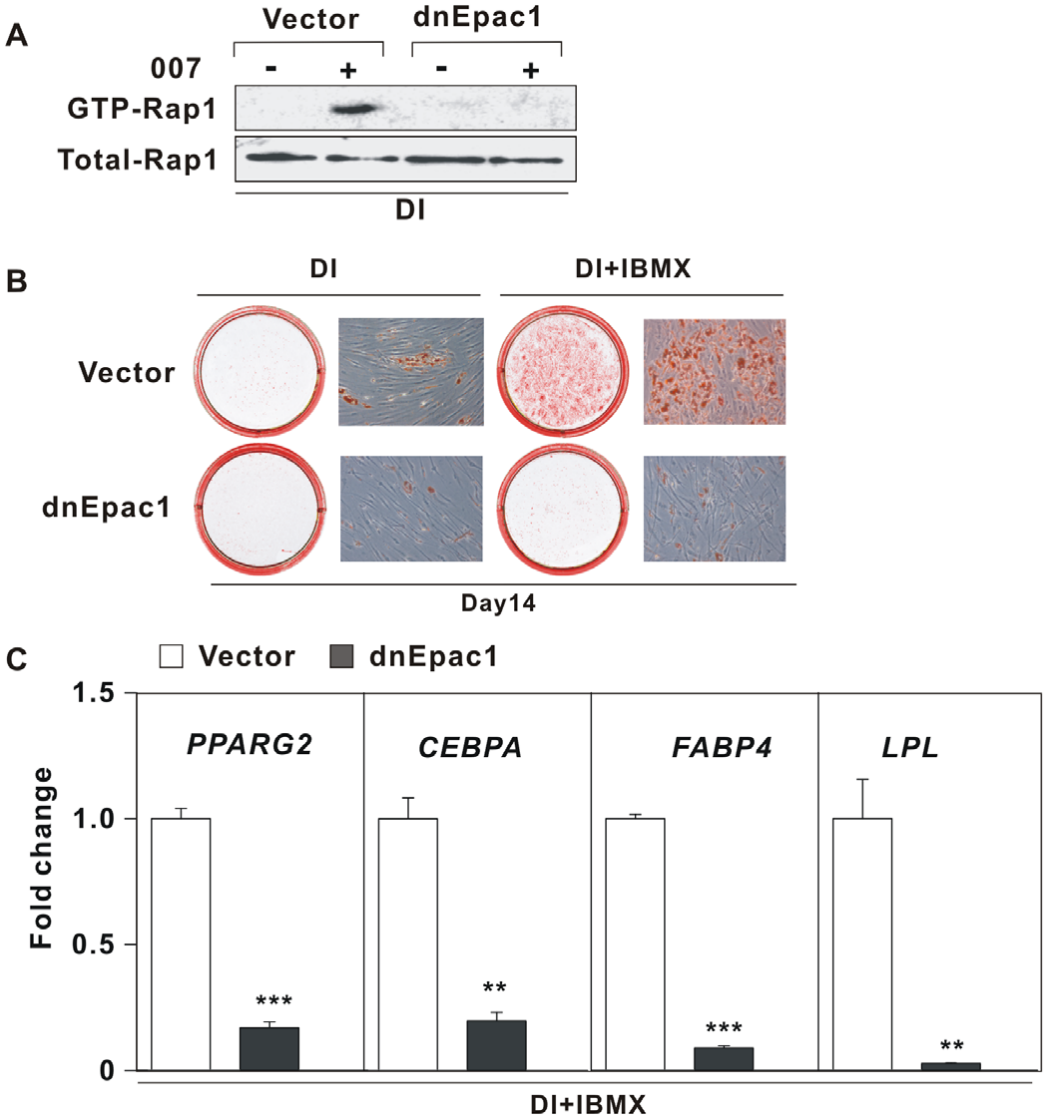
Dominant negative Epac1 attenuates adipocyte differentiation of hMADS cells. hMADS cells were infected with retroviruses expressing a dominant-negative form of Epac1 (dnEpac1) or an empty vector and grow to confluence. (A) GTP-bound Rap1 was measured by a Rap1 activation pull-down assay. (B) Cells were induced to differentiate with Dex, insulin, IBMX and Rosi as described in “Materials and Methods”. On day 14, cells were stained with Oil-Red-O and photographed. The photographs and micrographs shown are representative of 4 independent experiments. (C) *PPARG2*, *CEBPA*, *FABP4* and *LPL* mRNA levels were determined by RT-qPCR. Expression was normalized to cells transduced with empty vector. Significant differences are indicated by asterisks, **p<0.01, ***p<0.001, n = 6.

To substantiate the importance of Epac in 007-mediated Rap-activity in hMADS cells, we transduced hMADS cells with a retroviral vector expressing a dominant negative Epac1 (dnEpac1) or an empty vector. As predicted, 007 failed to activate Rap1 in cells expressing dnEpac1, but increased the levels of GTP-associated Rap1 in cells transduced with the empty vector (Fig. 4A). To further corroborate the importance of Epac1 in cAMP-stimulated adipose conversion, hMADS cells expressing dnEpac1 or the empty vector were induced to differentiate by the hormonal differentiation cocktail including IBMX. As shown in Fig. 4B, hMADS cells expressing dnEpac1 did not accumulate lipids, whereas cells expressing the empty vector rounded up and accumulated lipids as evidenced by Oil-Red-O staining. The lack of an adipogenic phenotype in cells expressing the dnEpac1 was verified by real-time PCR analysis demonstrating a significantly reduced expression of adipogenic markers compared to cells expressing the empty vector (Fig. 4C). Taken together, these results demonstrate that Epac1 signaling is required for IBMX induced adipogenesis of hMADS cells.

### Epac activation enhances adipocyte differentiation of hMADS cells when ROCK is inhibited

**Figure 5:**
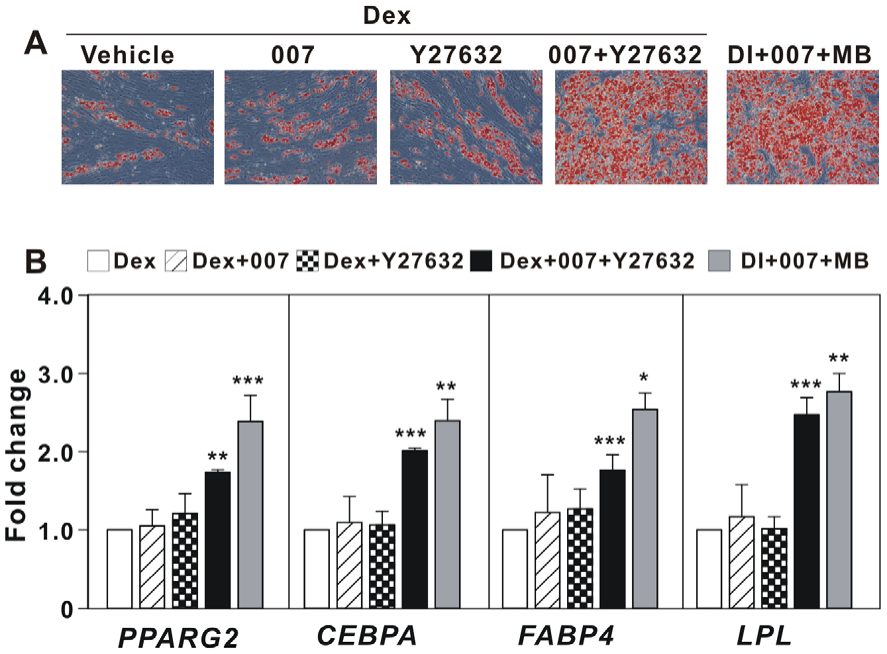
Effects of ROCK inhibitor Y-27632 on adipocyte differentiation of hMADS cells. Two-day post-confluent hMADS cells were induced to differentiate with 1 µM Dex in the presence of 200 µM 007, 10 µM Y-27632 either separately or in combination from day 0 to day 3. At the same time, another group of cells were treated with 1 µM Dex, 0.86 µM insulin (DI) in the presence of 200 µM 8-pCPT-2′-O-Me-cAMP (007) and 100 µM 6-MB-cAMP (MB). From day 3 to day 9, the medium contained 0.5 µM Rosi and 0.86 µM insulin. The medium was changed every second day. On day 14, (A) cells were stained by Oil Red O and photographed; (B) Total RNA was isolated and the expression of *PPARG2*, *CEBPA*, *FABP4* and *LPL* was determined by RT-qPCR. Expression was normalized to the value of Dex treated cells. Significant differences are indicated by asterisks, *p<0.05, **p<0.01, ***p<0.001, n = 9.

In murine preadipocytes Epac activation promotes adipocyte differentiation by counteracting the decrease in IGF-1/insulin signaling mediated via the PKA-dependent inhibition of Rho/ROCK activity [44]. To examine if the same mechanism operates in hMADS cells, we treated hMADS cells with the ROCK inhibitor Y-27632 [42] in the presence or absence of 007. As shown in Fig. 5, administration of Y-27632 in the presence of Dex did not stimulate adipogenesis. Similarly, treatment with the Epac activator 007 in the presence of Dex did not promote adipogenesis. However, treatment of hMADS cells with Y-27632 in combination with 007 significantly enhanced adipocyte differentiation, as demonstrated by both Oil Red O staining and adipocyte marker genes expression (Fig. 5A and B).

### Effects of PPARs agonists and long-chain fatty acids on adipocyte differentiation of hMADS cells

**Figure 6:**
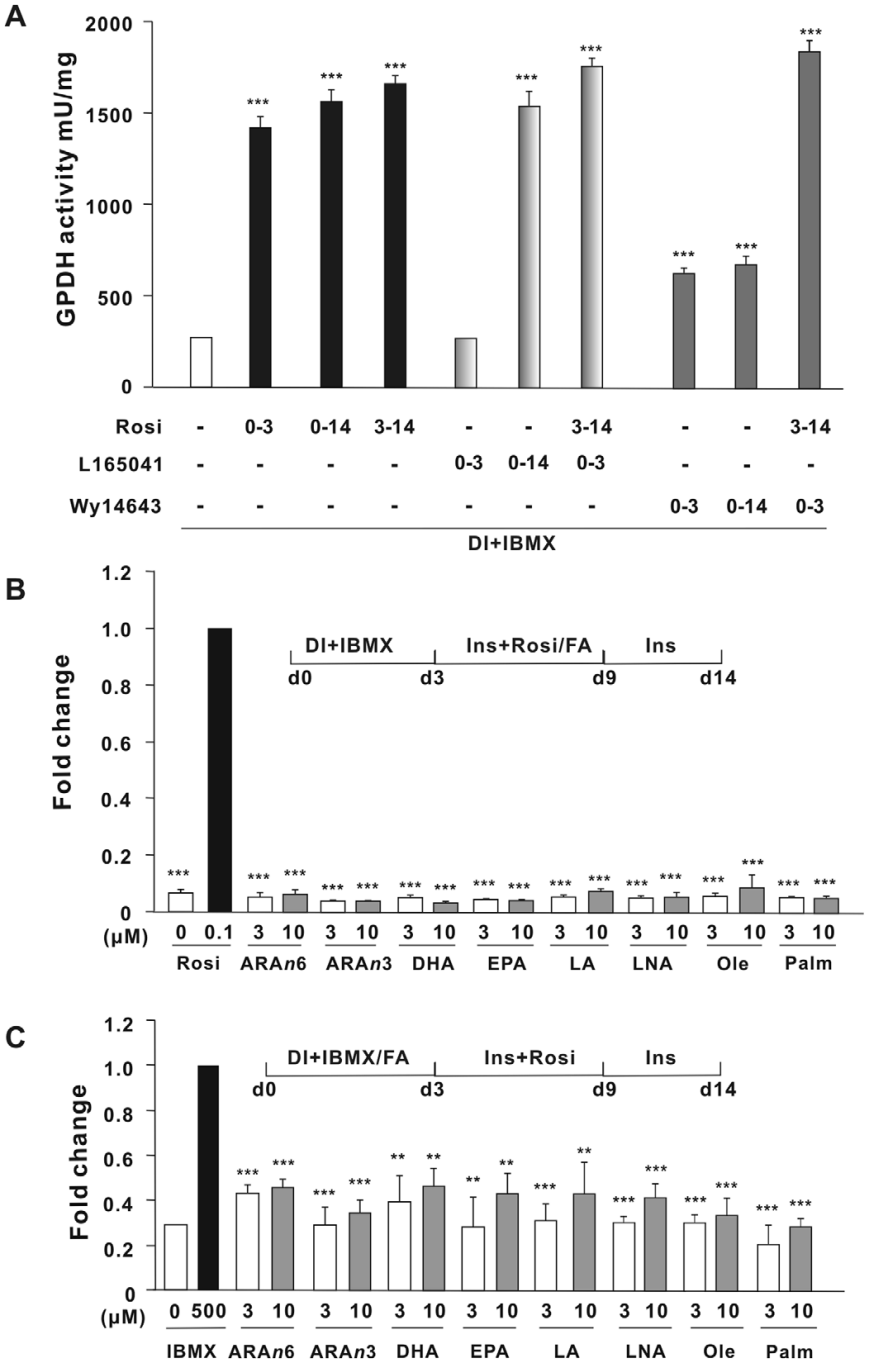
Effects of PPAR agonists and long-chain fatty acids on adipocyte differentiation of hMADS cells. Two-day post-confluent hMADS cells were maintained in serum-free DMEM/Ham's F12 medium in the presence of 1 µM Dex, 0.5 mM IBMX, and 0.86 µM insulin. From day 3, the medium consisted of DMEM/Ham's F12 with 0.86 µM insulin and was changed every second day. In addition, (A) 0.5 µM PPARγ agonist rosiglitazone (Rosi), 0.5 µM PPARδ agonist L165041, or 20 µM PPARα agonist Wy14643 was added for the indicated time periods; Significant differences relative to control are indicated by asterisks, ***p<0.001, n = 6. (B) Rosi, *n*-6 arachidonic acid (ARA*n*6), *n*-3 arachidonic acid (ARA*n*3), docosahexaenoic acid (DHA), eicosapentaenoic acid (EPA), linoleic acid (LA), α-linoleic acid (LNA), oleic acid (Ole), and palmitic acid (Palm) dissolved in DMSO were added at the indicated concentrations from day 3 to day 9. GPDH activities were determined on day 14. In panel B, data presented were normalized to Rosi treated cells. Significant differences relative to Rosi are indicated by asterisks, ***p<0.001, n = 6. C) Two-day post-confluent hMADS cells were maintained for 3 days in serum-free DMEM/Ham's F12 medium in the presence of 1 µM Dex and 0.86 µM insulin supplemented with either 0.5 mM IBMX or various fatty acids as indicated. From day 3 to day 9, 0.5 µM Rosi was then added and GPDH activities were determined on day 14. Significant differences relative to IBMX-treated cells are indicated by asterisks, **p<0.01, ***p<0.001, n = 6.

PPARs play critical roles in the development and metabolic regulation of adipose tissue, and the cAMP-dependent synthesis of endogenous PPARγ ligand(s) has been shown to be crucial during the initial stage of adipocyte differentiation of murine preadipocytes [26], [45]. To initially characterize the possible temporal contribution of the different PPAR isoforms, including PPARα and PPARδ, in adipogenesis, we treated two day-post-confluent hMADS cells (defined as day 0) with the hormonal cocktail including dexamethasone, IBMX and insulin in the absence or presence of selective PPAR activators for different periods of time. To monitor adipocyte differentiation we measured the increase in glycerol-3-phosphate dehydrogenase (GPDH) activity on day 14. GPDH is, a robust indicator of adipocyte differentiation and required for triglyceride synthesis [46]. As expected, treatment with rosiglitazone stimulated a robust increase in GPDH activity (Fig. 6). Treatment with rosiglitazone during the initial stage of differentiation, i.e. during day 0–3 was sufficient to elicit a robust increase in GPDH activity, which only increased slightly by prolonged treatment. Furthermore, treatment with rosiglitazone from day 3–14 also induced robust differentiation as determined by the increased GPDH activity. A weak differentiation was observed in cells treated with the PPARα agonist Wy14643 regardless of the exposure time. By contrast, in murine preadipocytes treatment with PPARα agonists elicits a robust induction of adipocyte differentiation [20], a difference that may at least in part relate to the different affinity of the agonist with respect to mouse and human PPARα [47], [48]. Of note, cells treated chronically with the PPARδ agonist L165041 also exhibited high levels of GPDH activity (Fig. 6A). Whether this reflected the ability of L165041 to function as a weak PPARγ agonist or whether activated PPARδ was able to induce expression of PPARγ in human cells as described for murine preadipocytes [22], [49] remains to be elucidated. However, the fact that treatment with L165041 during the first three days of the differentiation process did not induce adipocyte differentiation similar to treatment with the selective PPARγ agonist rosiglitazone indicates that L165041 did not promote adipocyte by direct activation of PPARγ. Collectively, these results indicate that both PPARγ and PPARδ agonists promote adipocyte conversion of hMADS cells consistent with the expression of their cognate receptors in confluent undifferentiated hMADS cells [50].

**Figure 7:**
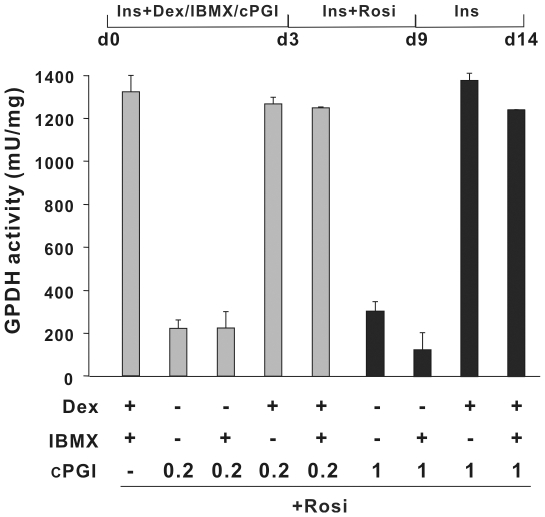
Carbaprostacyclin rescues adipocyte differentiation in the absence of other cAMP elevating agents. Two-day post-confluent hMADS cells were maintained in induction media with 0.86 µM insulin in the presence or absence of 1 µM Dex, 0.5 mM IBMX or 0.2 or 1 µM carbaprostacyclin (cPGI) as indicated from day 0 to day 3. Thereafter, 0.5 µM rosiglitazone was added until day 9. GPDH activities were determined on day 14.

Long-chain fatty acids and a plethora of oxygenated fatty acid derivatives bind to and activate the three members of the PPAR family [23], [27], [29], [30] and promote adipocyte differentiation of murine preadipocytes [11]. We tested the ability of a series of long-chain fatty acids to promote adipogenesis of hMADS cells in the presence of the adipogenic inducers dexamethasone, IBMX and insulin. None of the tested fatty acids promoted adipocyte differentiation of hMADS cells as determined by GPDH activity (Fig. 6B). Arachidonic acid can serve as a substrate for cAMP-dependent generation of PPARγ ligands [24], [25], [45]. The *n*-6 PUFA arachidonic acid has further been shown to promote adipocyte differentiation of murine Ob1771 preadipocytes [13] via a cyclooxygenase-dependent conversion into PGI_2_ which by binding to the PGI_2_ receptor, IP-R, enhances cAMP production [13], [26], [31]. However, addition of arachidonic acid did not promote adipocyte differentiation of hMADS in the presence of IBMX, suggesting that arachidonic acid-dependent ligand production is absent or too limited in hMADS cells to support adipocyte differentiation and/or the addition of arachidonic acid in combination with a cAMP elevating agent led to the production of inhibitory prostaglandins as previously observed for 3T3-L1 cells [6], [26]. Furthermore, arachidonic acid was unable to substitute for IBMX and promote adipocyte differentiation of hMADS cells treated with DEX, insulin and rosiglitazone (Fig. 6C). Interestingly, even low concentrations of carbaprostacyclin (cPGI), a stable analog of PGI_2_, rescued adipocyte differentiation of hMADS cells in the absence of IBMX suggesting that PGI_2_ synthesis, release or action on IP-R is impaired in hMADS cells (Fig. 7). The results shown in Fig. 7 also demonstrate the requirement for dexamethasone to support adipocyte differentiation of hMADS cells. Taken together these results emphasize the need for cAMP signaling in concert with treatment with a PPARγ or PPARδ agonist to secure efficient adipocyte differentiation of human hMADS mesenchymal stem cells.

## Discussion

Adipose tissue-derived stem cells are a promising source of human stem cells for regenerative medicine because of their convenient isolation and extensive proliferative capacities *in vitro*. Identifying the molecular cues and regulators that drive differentiation *in vitro* is crucial for therapeutic use of these cells. hMADS cells are multipotent stem cells established from the stromal-vascular fraction of infant adipose tissue. These cells can be differentiated into adipocytes under serum free, chemically defined conditions. Moreover, once differentiated, hMADS cells are like human adipocytes able to secrete leptin and adiponectin [51]. As hMADS cells also express the various components involved in fatty acid metabolism and exhibit a lipolytic capacity similar to that of human adipocytes [52], these features underscore the utility of hMADS to serve as a good model for studying molecular mechanisms of adipocyte differentiation and functions.

cAMP signaling pathways are known to regulate a wide array of cellular processes by acting through its downstream effectors proteins PKA and Epac, and several studies have clearly demonstrated that the cAMP/PKA and cAMP/Epac pathways coexist in cells and function independently, but impinge on common molecular targets [35], [38], [53], [54]. However, the role of Epac in human adipocytes is still poorly understood. In the present study, we provide evidence that cAMP/PKA and cAMP/Epac pathways synergistically promote adipocyte differentiation of hMADS cells. Neither activation of PKA alone by 6-MB-cAMP nor activation of Epac by 8-pCPT-2′-O-Me-cAMP induced significant differentiation. However, when these two compounds were combined, differentiation was strongly enhanced. Inhibition of endogenous Epac1 activity by transfection with a dominant negative version of Epac1, dnEpac1, strongly attenuated the pro-adipogenic effect of cAMP. Similarly, like in murine preadipocytes, Epac activation enhanced adipocyte differentiation of hMADS cells in the presence of a ROCK inhibitor. Collectively, our results provide evidence that activation of both PKA- and the Epac-dependent pathways are necessary to promote adipocyte differentiation of hMADS cells. Contrasting murine preadipocytes, hMADS cells requires the addition of a PPARγ agonist for differentiation indicating that synthesis of endogenous PPARγ ligand(s) is insufficient to trigger adipogenesis *in vitro*. Of note, even in the presence of the powerful adipogenic inducers insulin, dexamethasone and rosiglitazone, efficient differentiation of hMADS cells still requires elevated intracellular levels of cAMP.

To further investigate the role of PPAR activation and the ability of various fatty acids to promote adipocyte differentiation of hMADS cells, we treated post-confluent hMADS cells with selective activators of PPARα, PPARδ and PPARγ. The PPARγ agonist rosiglitazone elicited a strong adipogenic response. Incubation with the PPARα agonist Wy14643 supported a modest increase in adipocyte differentiation as determined by GPDH activity, contrasting the situation in murine preadipocytes where PPARα agonists powerfully induce adipocyte differentiation [20]. A difference in binding affinity between human and murine PPARα may in part explain this difference [47], [48]. Recently, treatment of hMADS cell with the potent PPARα agonist GW7647 was shown to enhance fatty acid oxidation and GPDH activity, underscoring the possibility that differences in affinity may at least in part explain the lack of effect of Wy14643 on hMADS cells [55]. Of note, sustained incubation with the PPARδ agonist L165041 resulted in adipocyte differentiation to the same degree as incubation with rosiglitazone. Activation of PPARδ has been shown to induce expression of PPARγ in murine preadipocytes, and a similar mechanism may operate in the human hMADS cells. Once PPARγ expression has reached a certain threshold, stimulation of adipocyte differentiation may occur ligand-independent [24], [56]. However, it should be noted that whereas simultaneous administration of a PPARδ-selective ligand and IBMX significantly enhanced clonal expansion and the early expression of PPARγ in murine 3T3-L1 preadipocytes, this treatment only modestly promoted terminal differentiation [20]. Thus, the robust differentiation of hMADS cells treated with IBMX and a PPARδ agonist is remarkable and warrants further investigation.

Long-chain fatty acids have been shown to exhibit an adipogenic activity at least in part by activating PPARγ and PPARδ in murine preadipocytes in the absence of agents that elevates the intracellular levels of cAMP. In particular arachidonic acid has been shown to be adipogenic in conditions with low intracellular levels of cAMP, but also other *n*-6 PUFAs and *n*-3 PUFAs promote adipocyte differentiation of 3T3-L1 preadipocytes when the intracellular level of cAMP is low [6], [26], [57]. Arachidonic acid plays an additional role by giving rise to PGI_2_ *via* the cyclooxygenase pathway. After release from the cells PGI_2_ promotes differentiation of the murine preadipocytes by binding to the PGI_2_ receptor, IP-R, stimulating cAMP production [13], [26], [31]. In contrast to the effect on murine preadipocytes, none of these fatty acids promoted adipocyte differentiation of hMADS cells in the presence of a cAMP elevating agent. Furthermore, arachidonic acid was unable to substitute for IBMX and promote adipocyte differentiation of hMADS cells treated with DEX, insulin and rosiglitazone (Fig. 6C). The finding that addition of carbaprostacyclin (cPGI) a stable analog of PGI_2_ rescued adipocyte differentiation of hMADS cells in the absence of IBMX, but in the presence of insulin and rosiglitazone, indicates that insufficient amounts of PGI_2_ are synthesized by hMADS cells or PGI_2_-dependent signaling is abrogated even with addition of exogenous arachidonic acid despite the fact that IP-R, COX1 and COX2 are expressed in confluent hMADS cells (E.-Z. Amri; unpublished observation). Alternatively, arachidonic acid or other PUFAs may in hMADS cells be converted into prostanoids that inhibit adipocyte differentiation [6], [26], [57], [58]. Because addition of arachidonic acid did not rescue differentiation in the absence of a PPARγ ligand, this also indicates that hMADS cells have an insufficient capacity for producing endogenous PPARγ ligands from arachidonic acid.

In conclusion, our results demonstrate a pivotal role of cAMP signaling during initiation of adipocyte differentiation of hMADS cells requiring both PKA- and Epac-dependent signaling. cAMP signaling has been shown to induce production of endogenous PPARγ ligand(s) in murine preadipocytes [24]–[26], [45]. By contrast, whereas cAMP signaling is also required for differentiation in human mesenchymal hMADS cells, it does not induce sufficient synthesis of PPARγ ligand(s) to promote adipocyte differentiation even in presence of fatty acids that can serve as precursors for synthesis of PPARγ ligands. In 3T3-L1 cells, cAMP-dependent activation of PKA increases the activity of PPARδ promoting the early stages of adipocyte differentiation, but not terminal differentiation [22]. By contrast, combined treatment of hMADS cells with IBMX and a PPARδ agonist robustly induced terminal adipocyte differentiation. Furthermore, activated PKA also represses Rho-kinase activity, which allows adipocyte differentiation [44]. Finally, arachidonic acid, which in murine preadipocytes can enhance cAMP production via synthesis of PGI_2_, is unable to replace the addition of a cAMP-elevating agent in hMADS cells (Fig. 7). The fact that addition of the stable PGI_2_ analog carboprostacyclin (cPGI) rescued adipocyte differentiation in the absence of other cAMP-elevating agents points to impaired PGI_2_ synthesis, secretion and/or signaling via the PGI_2_ receptor in hMADS cells, further underscoring the difference between the human and the murine systems.

## Materials and Methods

### Chemicals

The cAMP analogs were purchased from Biolog (Bremen, Germany). All the other chemicals, except where specified were purchased from Sigma-Aldrich.

### Cell Culture and Differentiation

The establishment and characteristics of human multipotent adipose-derived stem (hMADS) cells have been described [17], [51], [59], [60]. Briefly, in the present study hMADS-2 cells were established from the pubic region fat pad of a 5-year old male donor after informed consent of the parents, and used between 16 and 35 passages corresponding to 35 to 100 population doublings. The protocol was approved by the Centre Hospitalier Universitaire de Nice Review Board.

hMADS cells were routinely cultured in Dulbecco's modified Eagle's medium (DMEM) (Lonza, Denmark) supplemented with 10% (v/v) fetal bovine serum (FBS), 2.5 ng/ml hFGF2 (Invitrogen), 60 µg/ml penicillin, and 50 µg/ml streptomycin, at 37°C in a humidified atmosphere containing 5% CO_2_. Medium was changed every other day. For adipogenic differentiation, hFGF2 was removed when cells reached confluence. Two-days-post-confluent (designated as day 0) cells were induced to differentiate in the presence of DMEM/Ham's F12 media (Lonza, Denmark) supplemented with 10 µg/ml transferrin, 0.2 nM triiodothyronine, 0.86 µM insulin, and 1 µM dexamethasone (Dex), 0.5 mM isobutylmethylxanthine (IBMX). After two days, the medium was changed (Dex and IBMX omitted), and 0.5 µM rosiglitazone (Novo Nordisk A/S, Denmark) was added. The media were then changed every other day and cells were harvested as indicated. 8-pCPT-cAMP (100 µM), 8-pCPT-2^′^-*O*-Me-cAMP (200 µM) (007), 6-MB-cAMP (100 µM) (MB), Rp-8-CPT-cAMPS (100 µM), Rp-8-Br-cAMPS (100 µM), carbaprostacyclin (0.2 or 1 µM)(cPGI)(Cayman Chemical, France), L165041 (0.5 µM)(Tocris Bioscience, France), Wy14643 (20 µM)(Cayman Chemical, France) and different fatty acids (3 or 10 µM in the presence of 1.5 or 5 µM human serum albumin) were added at the time stated in the legend of figures.

For Oil Red O Staining, cells were washed with PBS, fixed with 3.7% (w/v) formaldehyde for 1 h, rinsed twice with water, then stained for 1 h with Oil Red O [61]. After staining, cells were washed twice with water and photographed. Glycerol-3-phosphate dehydrogenase (GPDH) activities, a robust indicator of adipocyte differentiation required for triglyceride synthesis, were measured as described previously [54].

### Retroviral transduction

The dominant-negative Epac1 cDNA was kindly provided by Dr. Johannes L. Bos. pLXSN-hygro was kindly provided by Dr. O. A. MacDougald. The retroviral vectors encoding dominant-negative Epac1 (pLXSN-dnEpac1) has been described before [44], [62]. For transfection, phoenix-Ampho cells at 50% confluence were transfected with emptry vector (pLXSN) or pLXSN-dnEpac1 vector DNA using a standard calcium phosphate protocol. 48 h after transfection, virus supernatant were collected by centrifugation and filtered. Fifty percent confluent hMADS cells were transduced with virus supernatant diluted with one volume of fresh growth medium and 8 µg/mL polybrene. The following day, one successive cycle of transduction was performed to enhance the transduction efficiency. After that, cells were split and subjected to hygromycin B selection (400 µg/mL) (Calbiochem). After approximately 10 days, the selected clones were pooled and replated for differentiation.

### Real Time RT-PCR

Total RNAs were purified from cells using Trizol reagent (Invitrogen). 1 µg of total RNA, digested with RQ1 Dnase (Invitrogen), was reverse transcribed using MMLV reverse transcriptase (Invitrogen) and random hexamers (Amersham Pharmacia Biotech) at 37°C for 1 h. After cDNA synthesis, reactions were diluted with 50 µL of water and frozen at −80°C. Real-time quantitative PCR (qPCR) was performed in 20 µL reactions containing SYBR Green Mastermix, 1.5 µL of diluted cDNA and 300 nM of each primer using an Mx3000P real-time PCR instrument (Stratagene). Reaction mixtures were preheated at 95°C for 10 min, followed by 40 cylcles of 95°C for 15 s and 60°C for 30 s. PCR was carried out in 96-well plates and in duplicate. Primers were designed using Primer Experss 2.0 (Applied Biosystems) and the sequences are listed in Table S1 in supporting information. Target gene mRNA expression was normalized to the expression level of and endogenous house-keeping gene beta-2-microglobulin (B2M).

### Whole cell extracts and Western Blotting

Whole cell lysates were prepared in SDS sample buffer as described before [61]. Protein content was measured by Bradford method (Bio-Rad). Equal amount of protein was loaded for electrophoresis and proteins were transferred onto PVDF nitrocellulose membranes (Millipore). Equal loading/transfer was confirmed by Amido Black staining of membranes. Membranes were blocked for 1 h in TBS containing 5% nonfat dry milk. Incubation with primary and secondary antibodies was performed in TBS containing 5% nonfat dry milk for 1 h. Membranes were then washed in TBS and signals were visualized with enhanced chemiluminescence (ECL). The primary antibodies used were rabbit anti FABP4/aP2 antibody (Caymen Chemical) and mouse anti vimentin antibody (DAKO, Denmark A/S). Secondary antibody was horse-radish peroxidase-conjugated anti-rabbit or anti-mouse antibodies obtained from DAKO.

### Rap1 activation assays

Cells were stimulated for 15 min in medium with 200 µM 8-pCPT-2′-O-Me-cAMP or 100 µM 6-MB-cAMP or equal volume of water (vehicle) in the presence of Dex (1 µM) and insulin (0.86 µM). Then cells were lysed in Rap1 activation lysis buffer. Rap1 activation assays were performed in accordance with the protocol of the Rap1 activation assay kit (Upstate). Briefly, lysate was clarified by centrifugation and a portion of the cell lysate was reserved for analysis for total Rap1 content. 500 µL lysate were incubated with GST-tagged RBD of RalGDS pre-coupled to glutathione beads to specially pull down the GTP-bound form of Rap1. The proteins were eluted with SDS sample buffer and analyzed by SDS-PAGE and Western blotting using Rap1 antibodies (Upstate).

### Determination of PKA activity in cell lysates

Cells were stimulated for 15 min with 200 µM 8-pCPT-2′-O-Me-cAMP/100 µM 6-MB-cAMP or equal volume of water in media containing Dex (1 µM) and Insulin (0.86 µM). Cells were washed in ice-cold phosphate-buffered saline, and lysed in 0.5 ml of 50 mM potassium phosphate buffer (pH 7.0) with 1 mM EGTA, 0.3 mM EDTA, 2 mM 1,4-dithioerythriol (DTE), 0.15% Triton X-100, and Complete protease inhibitor cocktail (Roche). Lysates were snap-frozen in liquid nitrogen and thawed immediately before the assay of kinase activity as described previously [44]. The blank value was determined by incubating in the presence of 100 nM of the inhibitor peptide from the heat-stable PKA inhibitor.

### Statistics

All experiments were performed in duplicate or triplicate and repeated three or four times. The data are presented as means ± S.D. Analysis of variance was performed by two-tailed Student's *t* test. Differences were considered statistically significant when *p* was<0.05.

## Supporting Information

Figure S1 **Effects of PKA inhibitors on adipocyte differentiation of hMADS cells.** Two-day post-confluent hMADS cells were maintained in induction media with 0.86 µM insulin, 1 µM Dex, 200 µM 8-pCPT-2′-O-Me-cAMP (007) and 100 µM 6-MB-cAMP (MB) in the presence or absence of 100 µM Rp-8-CPT-cAMPS and 100 µM Rp-8-Br-cAMPS (Rps) as indicated from day 0 to day 3. From day 3 to day 9, the medium contained 0.5 µM Rosi and 0.86 µM insulin. The medium was changed every second day. On day 14, (A) Cells were stained by Oil Red O and photographed; (B) Total RNA was isolated and the expression of *PPARG2*, *CEBPA*, *FABP4* and *LPL* was determined by RT-qPCR. Expression was normalized to the value of DI+007+MB treated cells. Significant differences are indicated by asterisks, *p<0.05, **p<0.01, ***p<0.001, n = 9.
(TIF)

Table S1 **List of real-time RT-PCR primer sequences.**
(DOCX)
