# Supplementary figures and images for: Activation of Protein Kinase A and Exchange Protein Directly Activated by cAMP Promotes Adipocyte Differentiation of Human Mesenchymal Stem Cells
Source: PLoS One. 2012 Mar 27;7(3):e34114. doi: 10.1371/journal.pone.0034114 (PMC3313974; doi:10.1371/journal.pone.0034114)

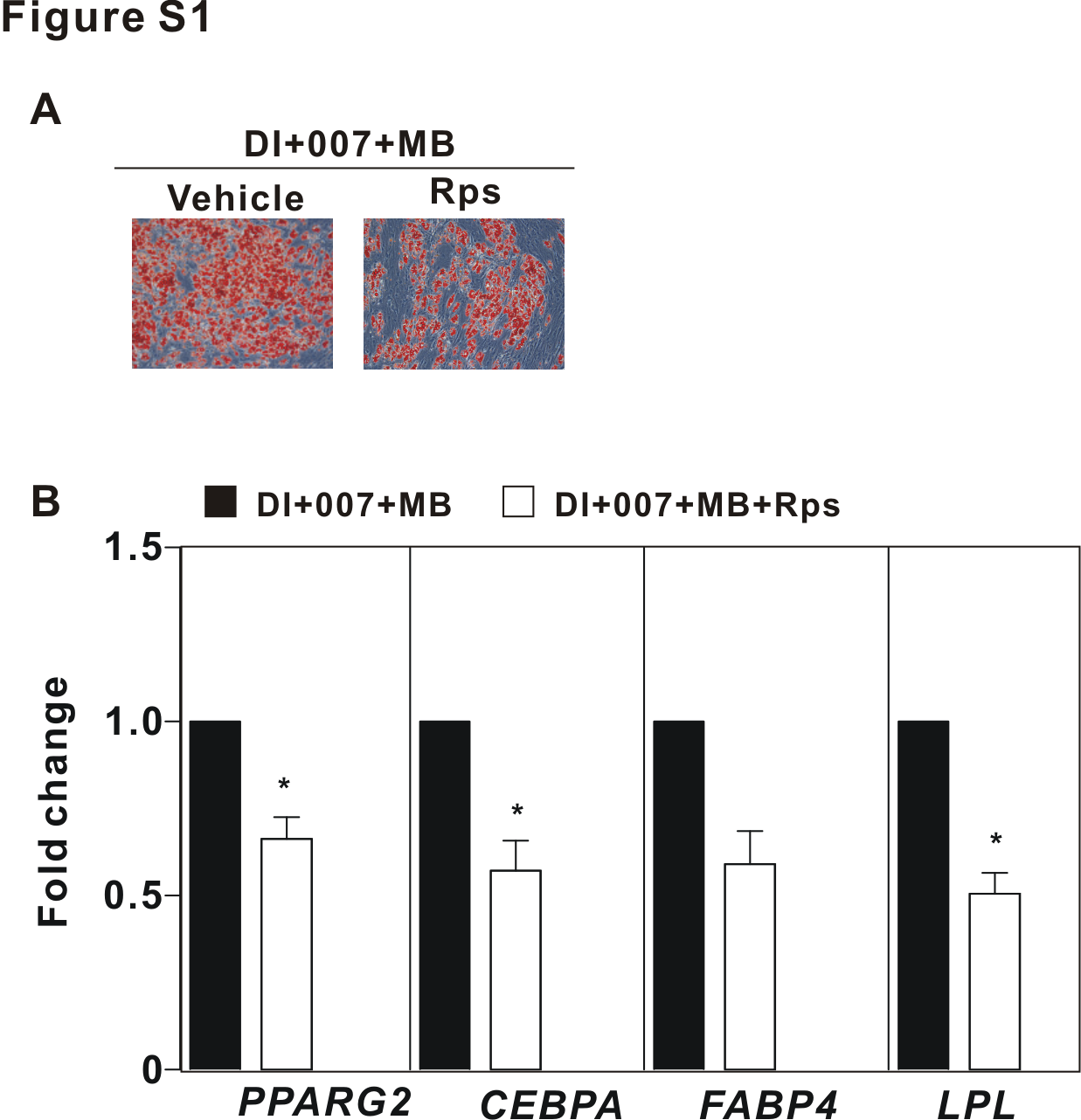

Supplement: Figure S1 — Effects of PKA inhibitors on adipocyte differentiation of hMADS cells. Two-day post-confluent hMADS cells were maintained in induction media with 0.86 µM insulin, 1 µM Dex, 200 µM 8-pCPT-2′-O-Me-cAMP (007) and 100 µM 6-MB-cAMP (MB) in the presence or absence of 100 µM Rp-8-CPT-cAMPS and 100 µM Rp-8-Br-cAMPS (Rps) as indicated from day 0 to day 3. From day 3 to day 9, the medium contained 0.5 µM Rosi and 0.86 µM insulin. The medium was changed every second day. On day 14, (A) Cells were stained by Oil Red O and photographed; (B) Total RNA was isolated and the expression of PPARG2, CEBPA, FABP4 and LPL was determined by RT-qPCR. Expression was normalized to the value of DI+007+MB treated cells. Significant differences are indicated by asterisks, *p<0.05, **p<0.01, ***p<0.001, n = 9. (TIF) [file pone.0034114.s001.tif]
