# Supplementary material for: Activation of Protein Kinase A and Exchange Protein Directly Activated by cAMP Promotes Adipocyte Differentiation of Human Mesenchymal Stem Cells
Source: PLoS One. 2012 Mar 27;7(3):e34114. doi: 10.1371/journal.pone.0034114 (PMC3313974; doi:10.1371/journal.pone.0034114)
Supplement: Table S1 — List of real-time RT-PCR primer sequences. (DOCX) [file pone.0034114.s002.docx]

Supporting information

Table S1 List of real-time RT-PCR primer sequences

| Gene | Accession No. | 5’primer | 3’primer |
| --- | --- | --- | --- |
| B2M | NM_004048 | TGGCCTTAGCTGTGCTCG | GGATGGATGAAACCCAGACAC |
| CEBPA | NM_004364 | CCCTCAGCCTTGTTTGTACTGTATG | TTCGTGTTCCTAGGCAATGCT |
| PPARG2 | NM_015869 | CTCCTATTGACCCAGAAAGCG | CAAAGTTGGTGGGCCAGAAT |
| LPL | NM_000237 | AGCTATCCGCGTGATTGCA | ACTAGCTGGTCCACATCTCCA AG |
| FABP4 | NM_001442 | ATATGAAAGAAGTAGGAGTGGGCTTT | CCATGCCAGCCACTTTCC |
| RAPGEF3 | NM_001098531 | GCTGCTCTGGCCGGGA | GAATTGGGCATCTCGGTCCT |
| RAPGEF4 | NM_007023 | ATTAATGGACGCCTGTTTGC | CTCCTCAGGAACAAATCCA |
| RAP1A | NM_001010935 | CAGTGTATGCTCGAAATCCTGG | TGCTGTAAATTGCTCTGTCCCT |
| RAP1B | NM_001010942 | GAGGCGTTGGAAAGTCTGCT | CCTCATTGCTGTAAATTGCTCC |
| RAP2A | NM_021033 | TCGTCTACAGCCTCGTCAACC | GGCACTTTCTCATACCGCTTC |
| RAP2B | NM_002886 | AAACAAAGCCTCGGTAGACGA | CTGCGCCGCGTAGTTCAT |
| RAP2C | NM_021183 | AGGTAGTGGTGTTAGGGAGTGG | GCTCAGTTCCTGCGGTGTC |
